# P07-08 The City of Antibes' Prescription Sports plan, the first « Health Sports Centre » in the Alpes-Maritimes department

**DOI:** 10.1093/eurpub/ckac095.108

**Published:** 2022-08-29

**Authors:** Yannick Madrignac, Isabelle Plisson, Romain Melan

**Affiliations:** Ville d'Antibes, France

**Keywords:** adapted monitoring regular accompanying autonomy

## Abstract

Today, Antibes is obviously the « Health and Sport city ». Demographic and epidemiological studies reveal problems of ageing and isolation of elderly people; often they are subject to serious health consequences and triggering the loss of autonomy. Precariousness and social isolation also enhance the occurence or deterioration of certain health problems (cardiovascular diseases, cancers, depression, etc.). All these factors lead to a growth of long term illnesses.

Following a national law health system modernisation in 2017, the City of Antibes, the Hospital Centre and the Regional Medical and Sport Centre have set up a program called “Pass Form Santé” dedicated to “Prescription Sports”.

What does it consist of? Who are the beneficiaries? What are the means implemented?

This unique and innovative program in the Alpes Maritimes aims to help people with chronic diseases to improve their health and the way of taking medication. It is to guide them to an adapted physical practice of activity at a regular, secure and progressive way on the recommendation of an attending physician.

The City of Antibes has all the resources to enable the implementation of this program: multi-disciplinary local municipal establishments, well trained human resources in different adapted physical activities, and a substantial network of sport and medical-social partners. Since 2017, 135 people have joined the “Pass Form Santé” program. 47% of prescriptions came from liberal or specialist doctors, 31% from hospitals and 22% from the Medical-Sportive Centre of Antibes. The breakdown of pathologies shows a majority cases in cardiology and oncology.

Qualitative result on the beneficiaries' health is unequivocal. The program allows them to feel better physically and morally. This “Pass Form Santé” program perfectly meets their expectations and allows them to get closer to an autonomous practice or to join any associative sports network.

The city of Antibes, together with its partners, has known how to create this innovative program called “Pass Form Santé” in order to develop prescription sports. It serves as a common link between beneficiaries, the medical professionals and the associative sport networks in order to encourage people with a chronic disease to resume physical activity.

